# Supplementary material for: Cingulate microstimulation induces negative decision-making via reduced top-down influence on primate fronto-cingulo-striatal network
Source: Nat Commun. 2024 May 17;15:4201. doi: 10.1038/s41467-024-48375-1 (PMC11101474; doi:10.1038/s41467-024-48375-1)
Supplement: Supplementary file 1 — Supplementary Information [file 41467_2024_48375_MOESM1_ESM.pdf]

Supplementary Information
for
Cingulate microstimulation induces negative decision-making via reduced top-down influence on primate fronto-cingulo-striatal network

Authors: Satoko Amemori[1, 2+], Ann M. Graybiel[3], and Ken-ichi Amemori[1*+]

Affiliations:

[1]Institute for the Advanced Study of Human Biology (ASHBi), Kyoto University

[2]Japan Society for the Promotion of Science

[3]McGovern Institute for Brain Research, Department of Brain and Cognitive Sciences, Massachusetts Institute of Technology

+: Equally contributed.

This file contains:

14 Supplementary figures

Supplementary Discussion

Supplementary References

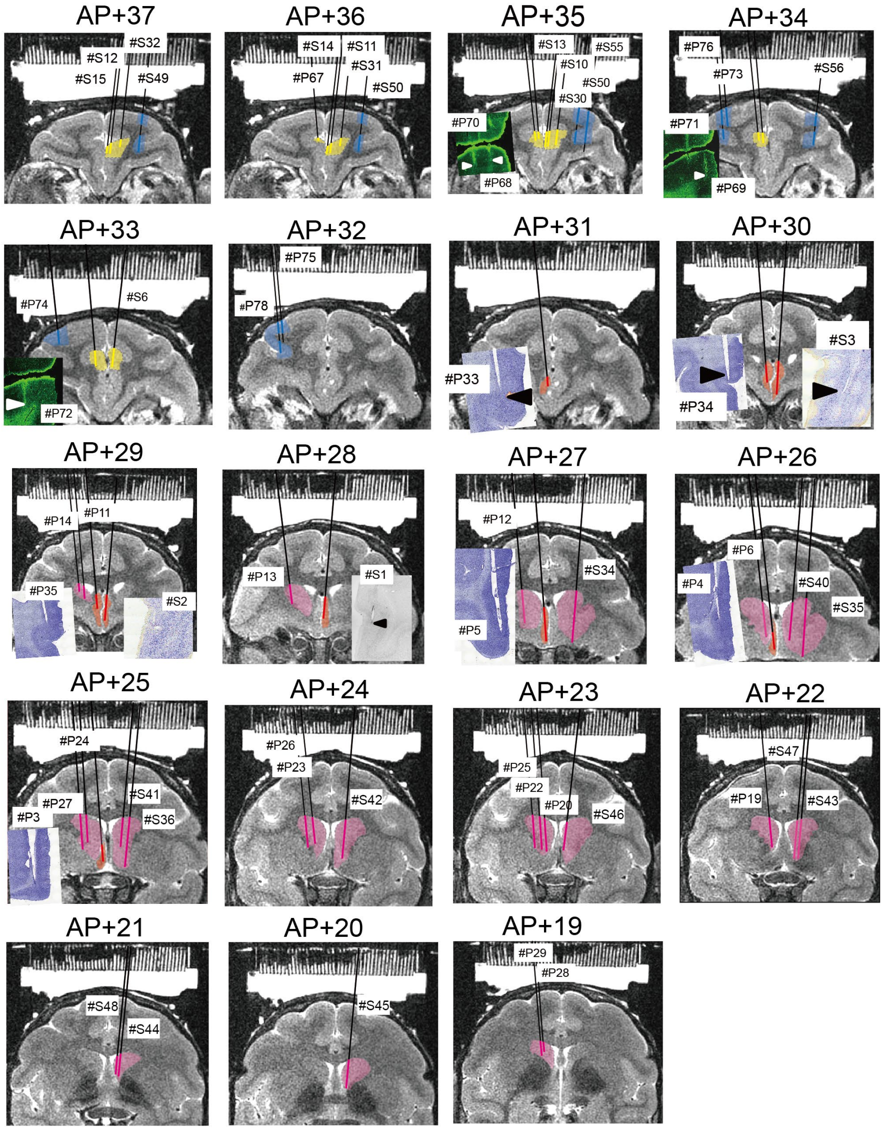

Supplementary Fig. 1. Histological reconstruction of electrode locations.

Histological reconstruction of our recording and microstimulation sites in the sgACC and pACC corresponds to areas 32, 10m, and 25. The electrode tracks of two monkeys were reconstructed according to the histological images consisting of brain sections stained with GFAP antibody (for monkey P) or nissl (for monkey S) (see Methods). The reconstructed tracks were mapped onto a coronal MRI image of monkey P. The corresponding histological images were also shown. The tip of an electrode was indicated with an arrowhead. The electrode identification number was written such that the electrode number followed a monkey name: S (monkey S) or P (monkey P). Images were shown from anterior to posterior in anterior-posterior (AP) coordination.

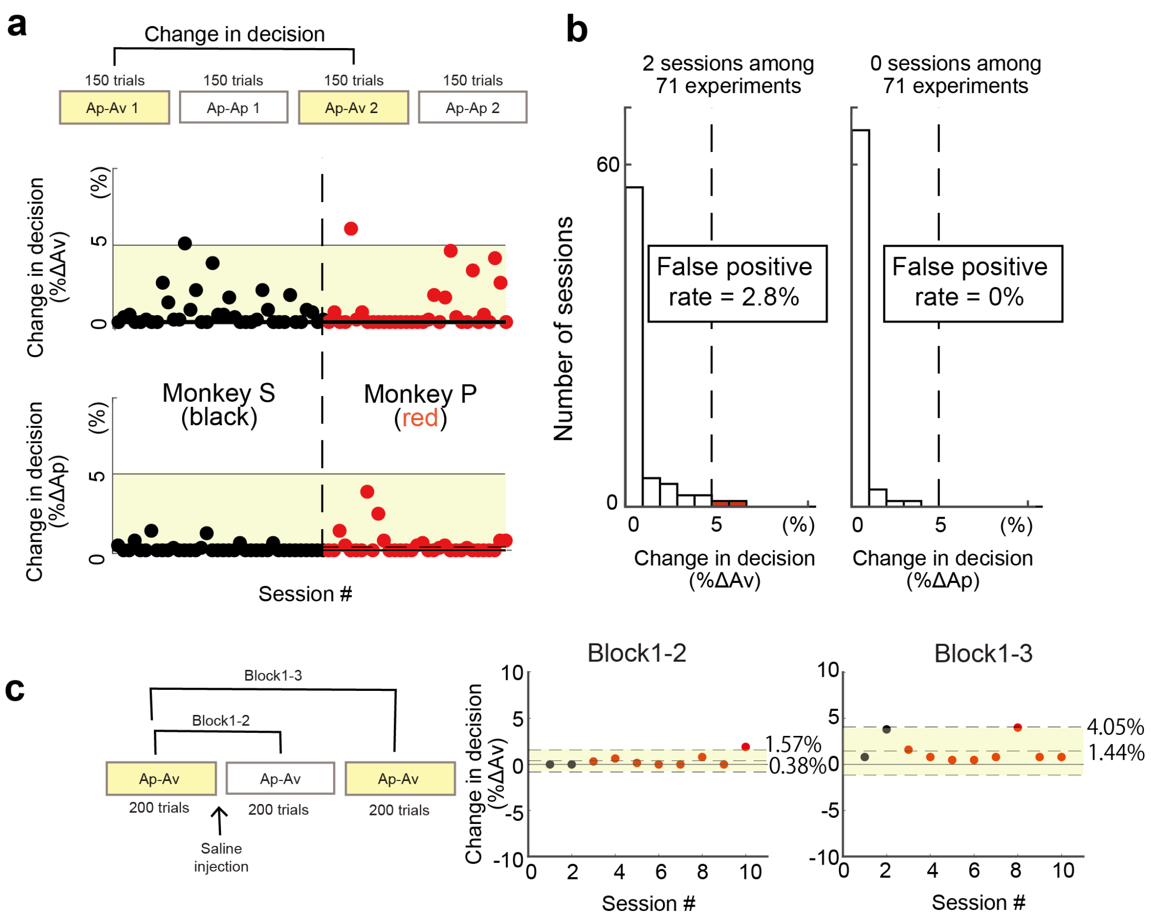

Supplementary Fig. 2. Stability of Ap-Av choices.

a, (top) Ap-Av1 block was separated from Ap-Ap2 block in recording sessions. (bottom) Change in Av decisions (%ΔAv, top) and Change in Ap decisions (%ΔAp, bottom) between Ap-Av1 and Ap-Av2 blocks were plotted along the session number for monkey S (black) and P (red). Yellow shading indicates the 5% discrimination threshold. **b,** The distribution of the change in decision frequencies quantified by %ΔAv (left) and %ΔAp (right). If we set the significance level to be 5% of the decision matrix, the false positive rate became 2.8% in Av choices. **c,** Stability of Ap-Av choices in no-stimulation sessions, illustrated with task block design (left) and choice behaviors in the 200 trials after the saline injection compared with those in the 200 trials before the injection (right).

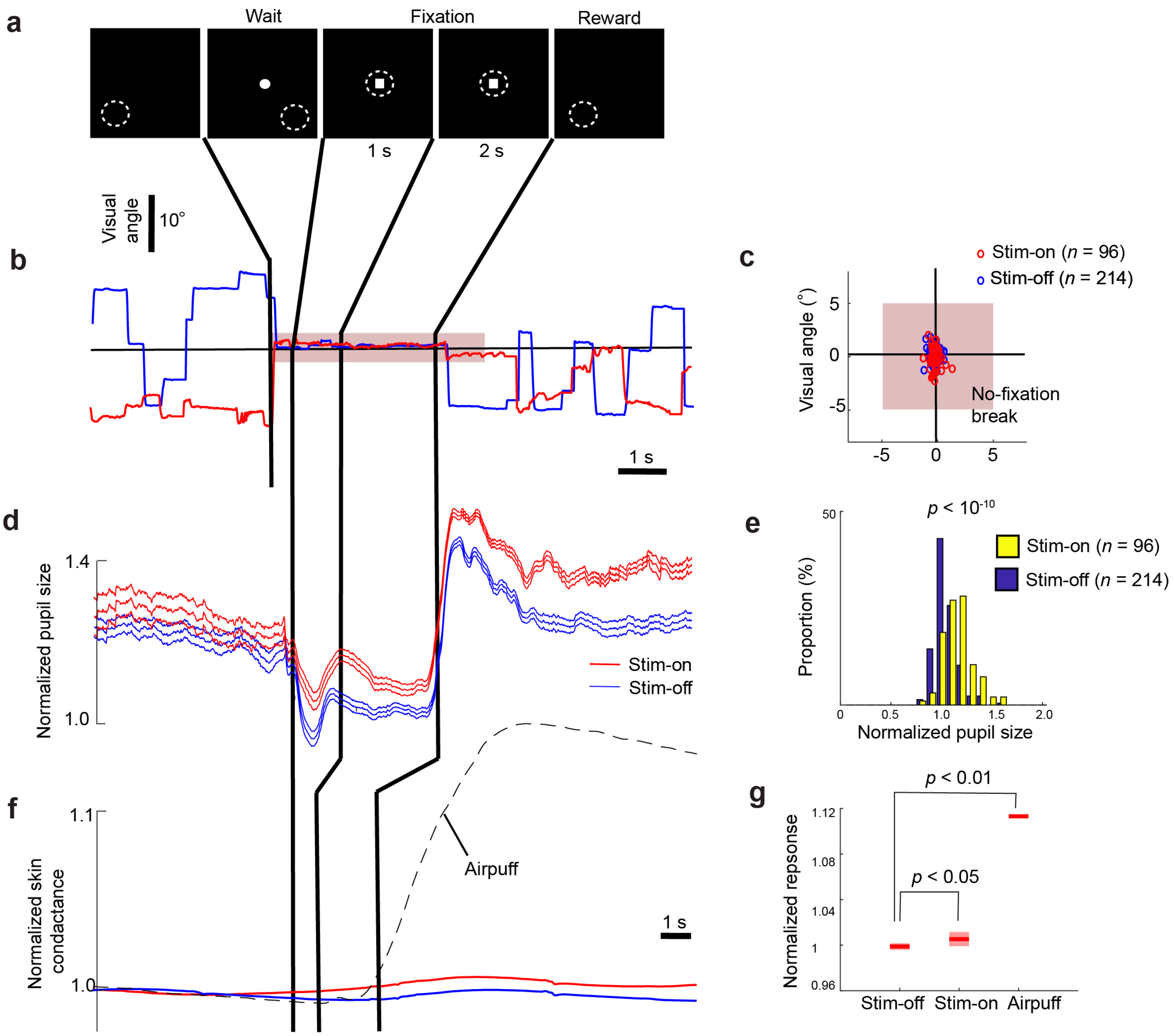

Supplementary Fig. 3. Physiological response induced by the sgACC microstimulation. Microstimulation of effective sgACC site did not induce eye movements but caused detectable pupillary dilation and skin conductance changes. The influence of the detectable physiological response on the decision was negligible compared to that of airpuff. **a,** Fixation task. **b,** Eye position (blue: x-axis, red: y-axis) around the central fixation point (black horizontal line) in a representative single trial. **c,** The mean eye position during the 3-s fixation period in the *Stim-on* (red) and *Stim-off* (blue) trials. Each panel represents the data from one session performed with stimulation at a negative effective site. A gray square denotes the fixation window (5° x 5°). Stimulation induced no fixation break. **d,** The mean (± SEM) pupil size for the *Stim-off* (red) and

Stim-on (blue) trials in one session. **e,** The distribution of mean pupil size during the 3-s fixation period in three individual sessions. Outside the fixation period, the pupil size estimated by video monitoring was not necessarily accurate due to eye movements, variation in depth of focus, or change in brightness. We thus compared the pupil sizes recorded during the fixation period. The mean of the distribution for the *Stim-on* (yellow bars) and *Stim-off* (blue bars) trials were compared by two-sampled t-tests. In the experiment, the pupils were significantly dilated by the stimulation ($P < 0.05$), suggesting a physiological response induced by the stimulation. **f,** Skin conductance averaged over the *Stim-on* (red) and *Stim-off* (blue) trials, compared with that responding to an unexpected airpuff delivered out of the task (dashed line). **g,** Significant changes in the skin conductance were induced not only by unexpected airpuff ($P = 0.003 < 0.01$, rank sum test), but also by the stimulation ($P = 0.0292 < 0.05$, t-test), suggesting that the stimulation itself produced some physiological responses, but it was very small compared to those induced by airpuff. Horizontal red lines indicate the mean and pink shading indicates SEM.

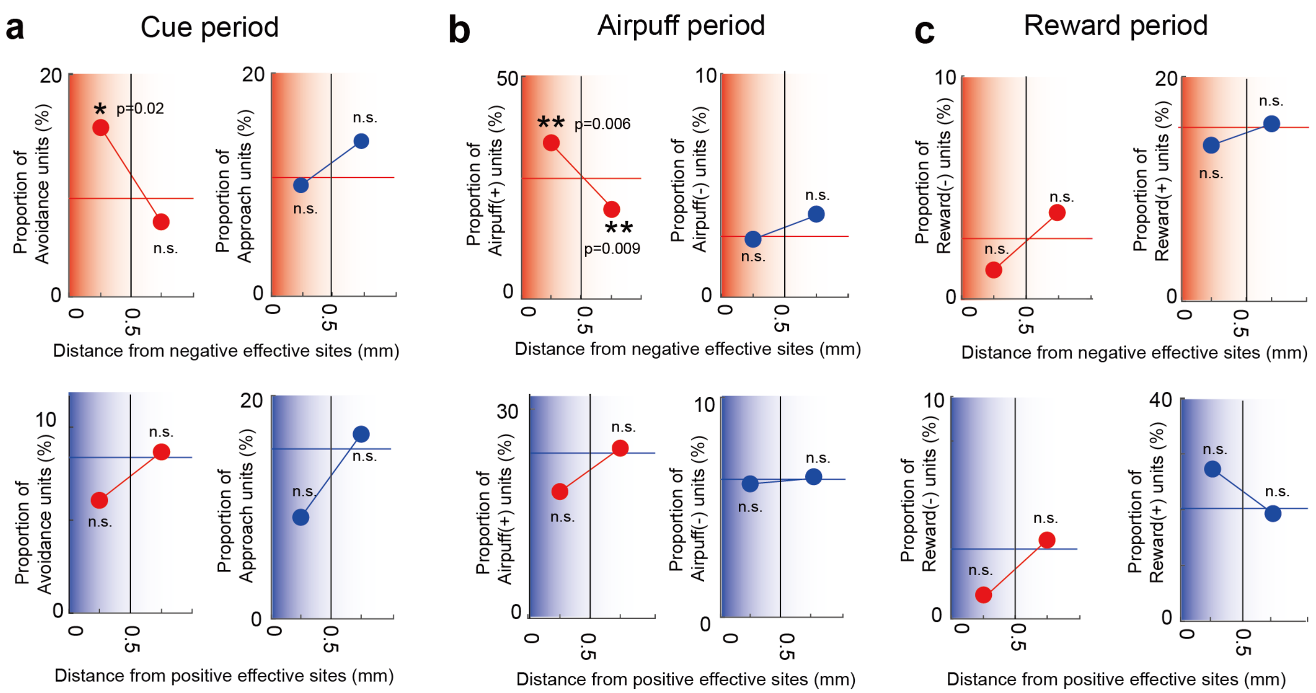

Supplementary Fig. 4. Avoudance and Airpuff (+) neurons were frequently recorded around the negative effective sites.

a, Distribution of each type of unit relative to negative and positive effective sites. Percentage of number of units/total number of recorded units were shown. On the left panel, the proportion of avoidance units (left) and approach units (right) were shown for 1-mm bins around negative (top) and positive (bottom) effective sites, respectively. **b,** The proportion of airpuff (+) units (left) and airpuff (–) units were shown around negative (top) and positive (bottom) effective sites, respectively. **c,** The proportion of reward (–) units (left) and reward (+) units (right) were shown around negative (top) and positive (bottom) effective sites, respectively. The asterisk indicates the bin in which the proportion of each unit was significantly different from that of aggregated over all tracks. P-value in Fisher's exact test was indicated.

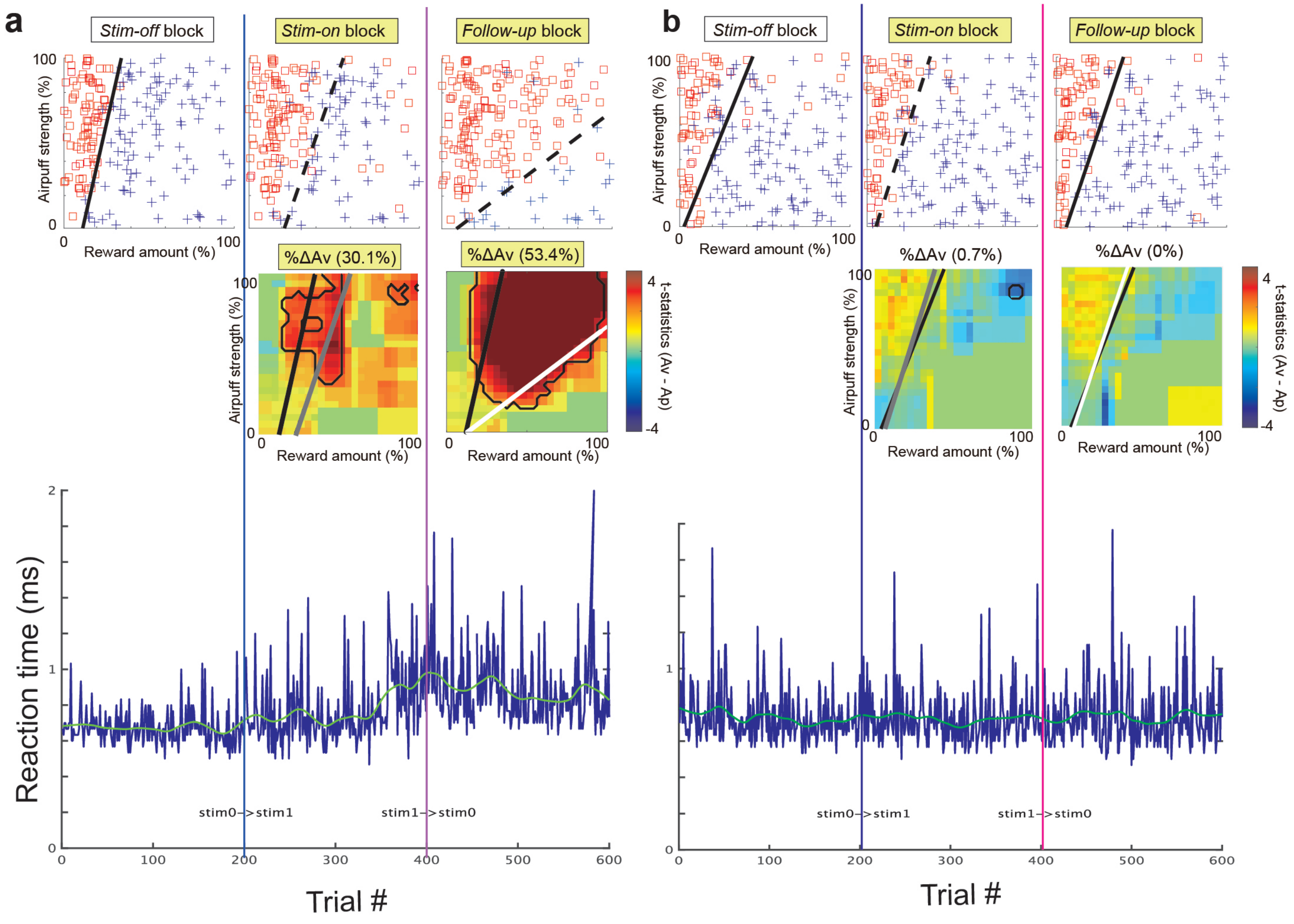

Supplementary Fig. 5. Microstimulation of Negative Effective Sites Persisted in a *Follow-up* Block.

a, Example of the stimulation-induced increase in Av choices persisted in the *Follow-up* block. Panels show, from left to right, the decision matrix for the first 200-trial block without stimulation (*Stim-off*), the second trial block during which stimulations (150 μA) were applied (*Stim-on*), and the third trial block after stimulations (*Follow-up*). Stimulation effect was measured by difference in the decision matrix (i.e., *Stim-on* – *Stim-off*). On the bottom, reaction times were plotted along the trial number. **b,** Persistent increase in Av choices was not observed in non-effective sites.

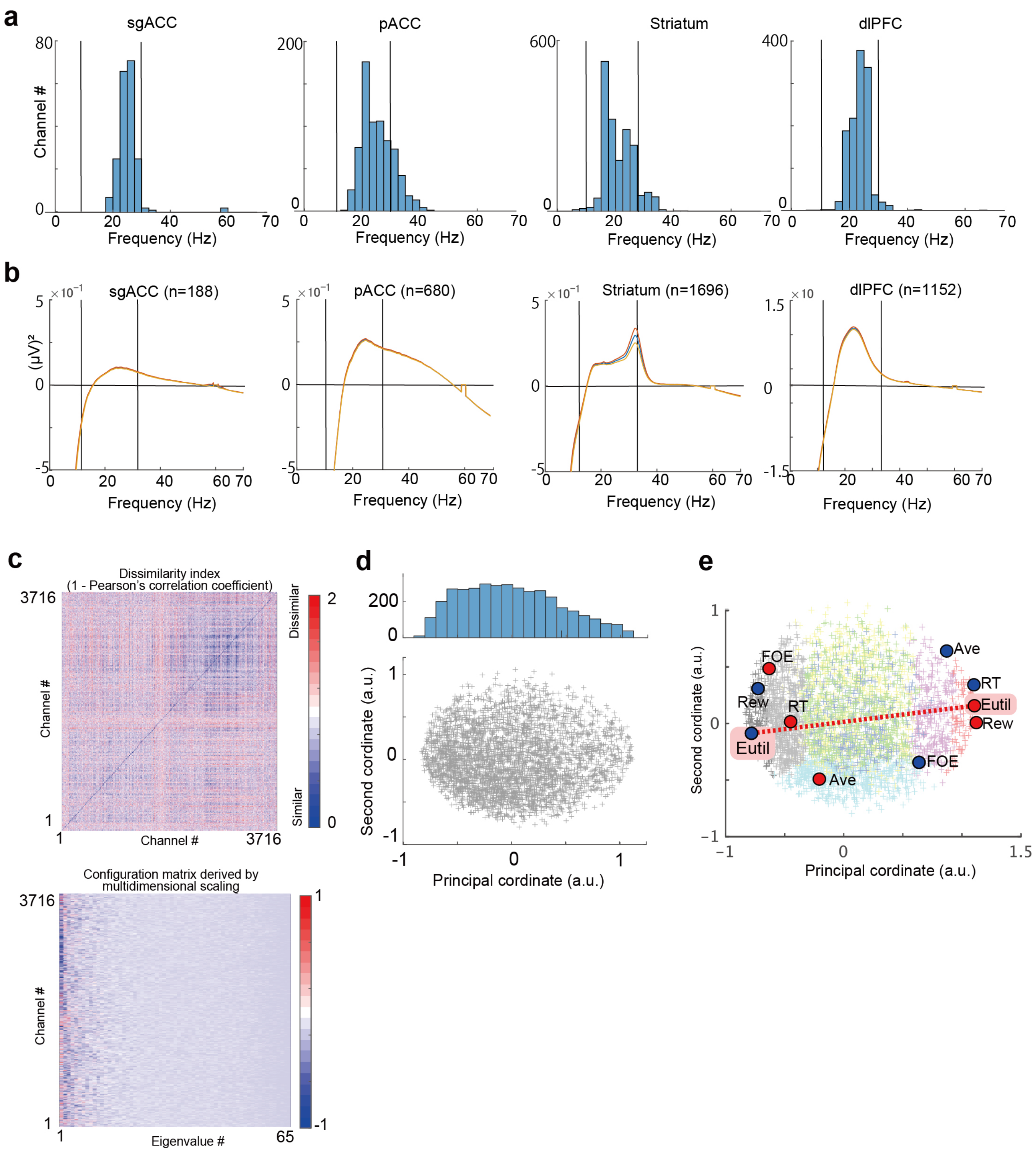

a
sgACC
pACC
Striatum
dlPFC
Channel #
Frequency (Hz)
b
sgACC (n=188)
pACC (n=680)
Striatum (n=1696)
dlPFC (n=1152)
(μV)²
Frequency (Hz)
c
Dissimilarity index
(1 - Pearson's correlation coefficient)
Channel #
Dissimilar
Similar
Configuration matrix derived by multidimensional scaling
Eigenvalue #
d
Second cordinate (a.u.)
Principal cordinate (a.u.)
e
FOE
Ave
RT
Rew
Eutil
Second cordinate (a.u.)
Principal cordinate (a.u.)

Supplementary Fig. 6. Procedure to analyze beta oscillations in recorded LFPs.

a, Distribution of peak frequencies for all LFPs recorded during *Stim-off* blocks in each region. The peak frequency of the maximum spectral power density in the baseline-subtracted LFP during the cue periods was derived. Many LFPs showed peak frequencies within the beta range (13-28 Hz) across all regions. **b,** Mean (± SEM) power spectrum of all *Stim-off* LFPs recorded from each region during the cue period after subtracting the fitted pink noise spectrum. **c,** Multi-dimensional scaling (MDS). The left panel shows the correlation distance matrix between pairs of all beta responses (D = [d_{ij}]). The color of each element shows the correlation distance (d_{ij} = 1 − r_{ij}), where r_{ij} is the cross-correlation between beta response *i* and response j recorded from each channel. The right panel shows a configuration matrix derived by the MDS function (cmdscale function of MATLAB). **d,** MDS plot of the LFPs. The bottom panel shows the plot along the principal and secondary coordinates, while the top panel shows the distribution along the principal coordinate. **e,** MDS, including behavioral parameters. The behavioral parameters we used are expected utility (Eutil), reaction time (RT), frequency of omission error (FOE), offered size of reward (Rew), and offered strength of aversive airpuff (Ave). Blue circle indicates a positive correlation with the parameter, and the red circle indicates a negative correlation. The first principle component (x-axis) distinguished positive and negative utility well.

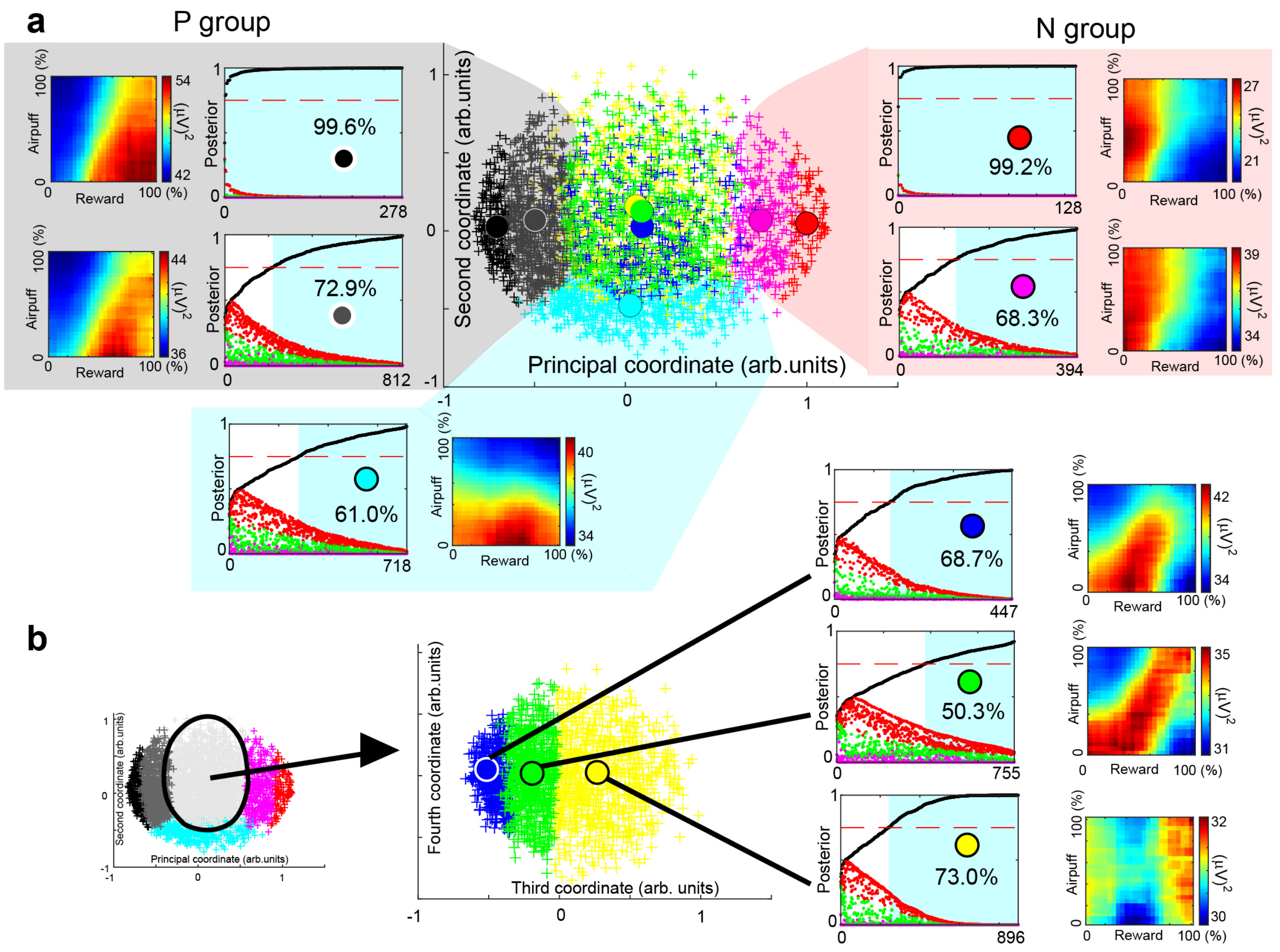

Supplementary Fig. 7. Posterior probabilities of eight groups and representational similarity analysis.

a, Posterior probability of each group. Posterior probabilities that quantify the degree of probability that each LFP was categorized as each group were calculated for each LFP. The MDS map was shown in the center as in Fig. 3d. For the P group that was shown on the left panels, the probability of being categorized as the P group was quantified for each LFP and shown for black and gray groups separately with their population activities on the left of each panel. Black dots in the right panel show the posterior probability of being categorized as P group, and the red, green and pink dots indicate the posterior for other groups with the second, third and fourth largest values. The X-axis indicates the number of channels, after which they were aligned by the posterior of the P group. For the N group shown on the right panels, the posterior of the N group (red and pink groups) was shown as black, and the red, green and pink dots indicate the posterior for other groups with the second, third and fourth largest values. Similarly,

on the bottom, the posterior of the cyan group was shown by black dots. For the panel of each posterior, the red horizontal and dotted line indicates 75%, and the blue area indicates the LFPs that exceeded the 75% threshold. The proportion of LFPs that exceeded the threshold was also shown for each panel. **b,** The third- and fourth-dimensional axes of the blue, green and yellow groups. In Fig. 3d, which showed the two-dimensional mapping of the MDS-based clustering, the blue, green and yellow groups overlapped among groups. In the third-dimensional axis, the LFPs of these groups were segregated, corresponding to the similarity of the population activity on the decision matrix (right panels). The posterior of being classified as each group was shown by black dots, and the red, green and cyan dots indicate the posterior for other groups with the second, third and fourth largest values.

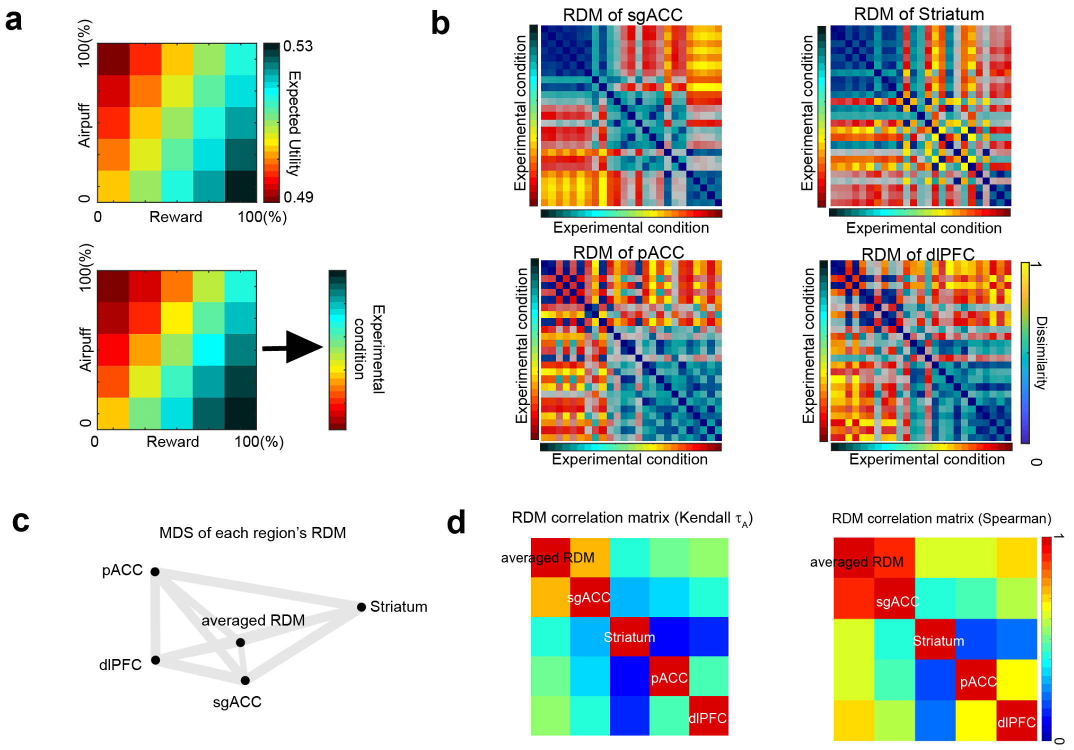

Supplementary Fig. 8. Posterior probabilities of eight groups and representational similarity analysis.

a, Experimental conditions of representational similarity analysis (RSA) to statistically compare how dlPFC, sgACC, pACC, and striatum differ in their responses to cue stimuli. Experimental conditions were derived by discretizing the offered sizes of the reward and air-puff into eight bins, resulting in 64 (= 8 × 8) cue stimuli. To determine the order of experimental conditions, we decoded the expected utility by regressing decision patterns in the decision-making model (top panel) and ranked the experimental conditions based on utility (bottom panel). **b,** Representational dissimilarity matrix (RDM) of each brain region of interest (i.e., sgACC, striatum, pACC and dlPFC). Magnitudes of beta oscillation were measured while the monkeys were exposed to these 64 experimental conditions. The regional activity pattern was estimated by the population activity of beta responses recorded for each experimental condition. A dissimilarity in representation was computed for each pair of activity patterns and put into a representational dissimilarity matrix (RDM). **c, d,** The dissimilarities between the activity patterns can be considered distances in the multivariate response space, and the RDM describes the geometry of the representation, serving as a signature that can be compared between different brain regions. To visualize the relationship among the representations of four brain regions, we

performed multi-dimensional scaling of the four RDMs (**c**) and the correlation analyses (Kendall's tau and Spearman's test) (**d**).

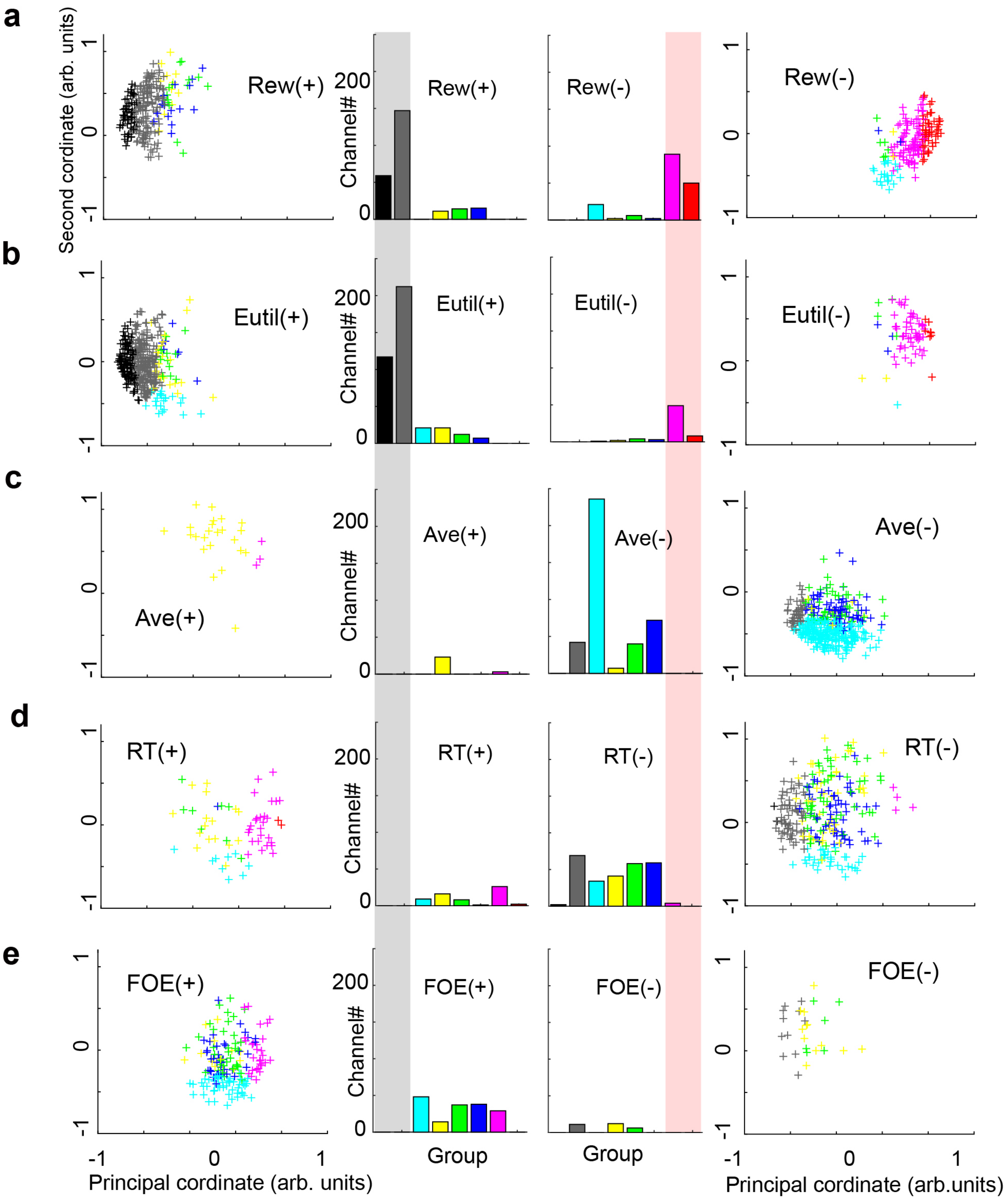
a
b
c
d
e
Rew(+)
Rew(-)
Eutil(+)
Eutil(-)
Ave(+)
Ave(-)
RT(+)
RT(-)
FOE(+)
FOE(-)
Second cordinate (arb. units)
Channel#
Group
Principal cordinate (arb. units)
Principal cordinate (arb.units)

Supplementary Fig. 9. Decoding beta representation for each group

The two panels on the left show MDS plots for the LFPs that the regression analysis classified as encoding different behavioral parameters: **a**, offered size of reward (Rew). **b**, offered strength of aversive airpuff (Ave). **c**, expected utility (Eutil). **d**, reaction time (RT), and **e**, frequency of omission error (FOE). Each cross represents an LFP, with the color corresponding to each group. The leftmost panels show the MDS plots for the LFPs exhibiting a positive (+) correlation with the behavioral parameter, and the rightmost panels show those exhibiting a negative (-) correlation. The two panels in the middle show the number of LFPs categorized for each group: the left shows LFPs exhibiting a positive correlation with the behavioral parameter, and the right shows those exhibiting a negative correlation. The color of each bar represents the group.

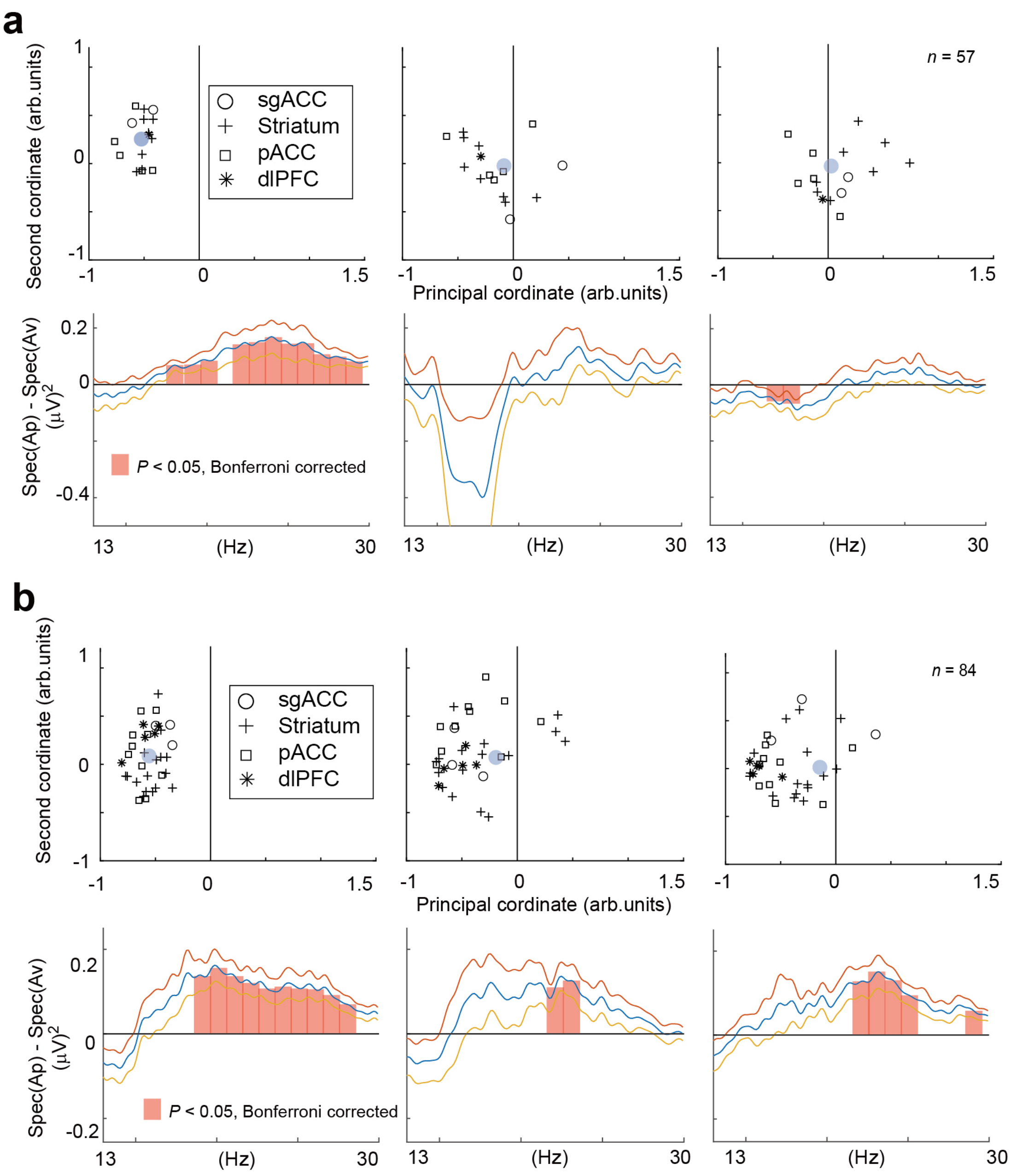

Supplementary Fig. 10. Stimulation effect on beta representation of P group

a, The top panels show MDS plots for LFPs recorded from channels categorized as the P group during the *Stim-off* block in negative effective sessions. The shape of the points represents the recorded locations, as shown in the inset. The left panel shows *Stim-off*, the middle panel shows *Stim-on* and the right panel shows *Follow-up* blocks. The bottom panels display the Ap-Av tuning index for the group, calculated by subtracting the spectrum for the Av choices from that for the Ap choices. We tested whether the Ap-Av tuning index significantly differs from zero for each

frequency, and the pink line indicates a significant difference (P < 0.05, t-test, Bonferroni corrected). **b,** The MDS space and their Ap-Av tunings for the LFPs are categorized as the P group in the *Stim-off* block in non-effective sessions. Each panel is illustrated as in **a**.

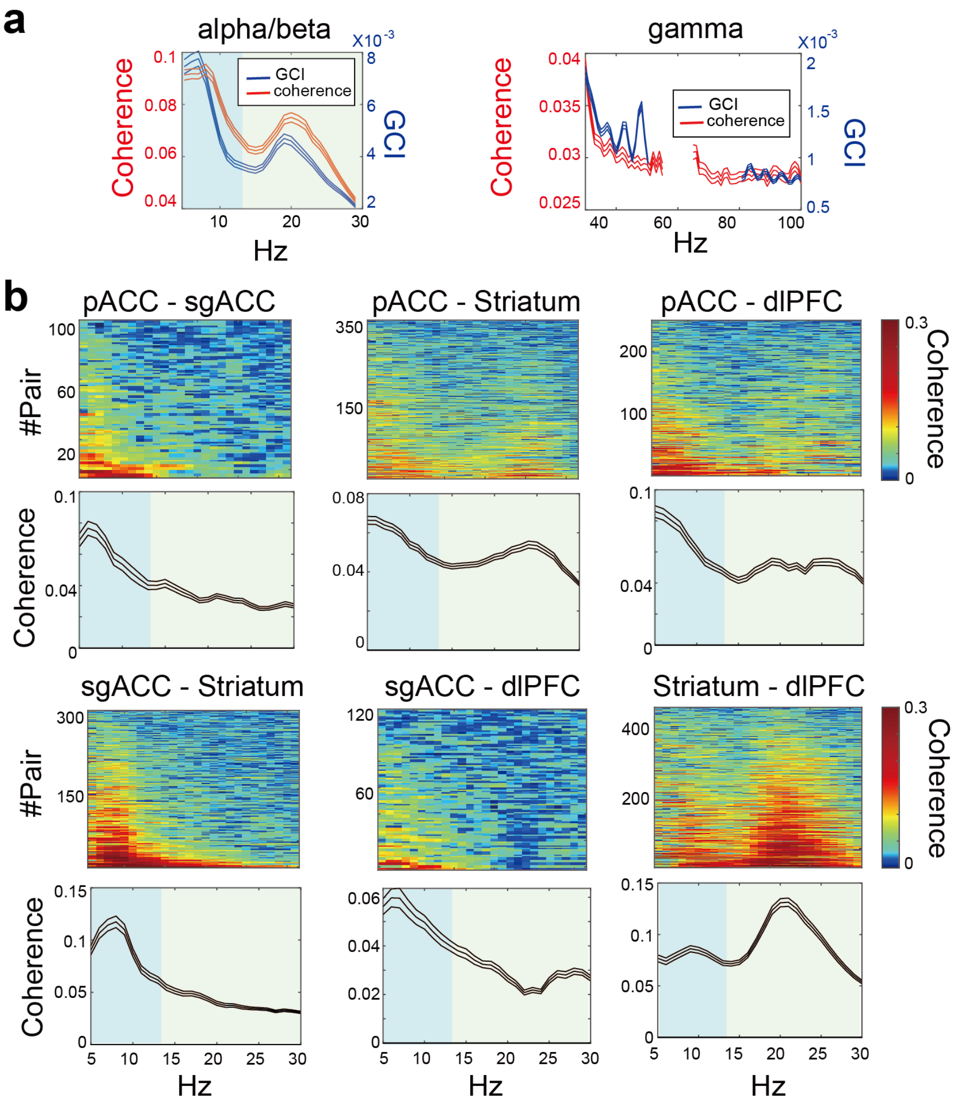

Supplementary Fig. 11. Coherence between regions for two monkeys.

a, The top panels show GCI (blue) and coherence (red) plots for all LFPs recorded from two monkeys during the *Stim-off* block in negative effective sessions. There were peaks in the alpha and beta ranges but not in the gamma range. Thus, we focused on alpha and beta ranges for further analysis. **b**, Coherence between each region during the *Stim-off* block in negative effective sessions. Coherence was plotted against each electrode pair.

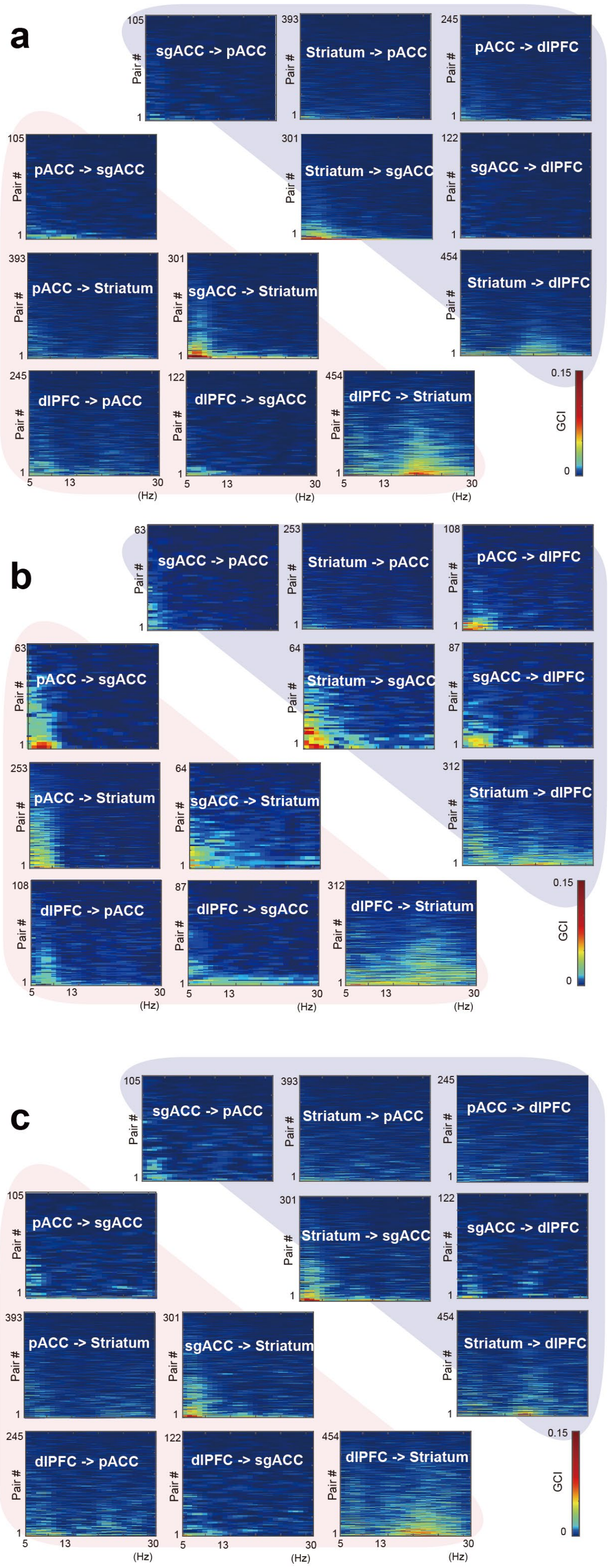

a
sgACC -> pACC
Striatum -> pACC
pACC -> dlPFC
pACC -> sgACC
Striatum -> sgACC
sgACC -> dlPFC
pACC -> Striatum
sgACC -> Striatum
Striatum -> dlPFC
dlPFC -> pACC
dlPFC -> sgACC
dlPFC -> Striatum
Pair #
(Hz)
GCI
0.15
0
b
sgACC -> pACC
Striatum -> pACC
pACC -> dlPFC
pACC -> sgACC
Striatum -> sgACC
sgACC -> dlPFC
pACC -> Striatum
sgACC -> Striatum
Striatum -> dlPFC
dlPFC -> pACC
dlPFC -> sgACC
dlPFC -> Striatum
Pair #
(Hz)
GCI
0.15
0
c
sgACC -> pACC
Striatum -> pACC
pACC -> dlPFC
pACC -> sgACC
Striatum -> sgACC
sgACC -> dlPFC
pACC -> Striatum
sgACC -> Striatum
Striatum -> dlPFC
dlPFC -> pACC
dlPFC -> sgACC
dlPFC -> Striatum
Pair #
(Hz)
GCI
0.15
0

Supplementary Fig. 12. Distributions of the change in DAI for two monkeys.

a, Granger Causality Indices (GCIs) in alpha and beta frequency ranges (5-30 Hz) were plotted for each direction of information flow, with the x-axis indicating frequency (Hz) and the y-axis indicating the index of each pair. The six panels in the top-right corner (blue shaded area) display the bottom-up influences (GCIs from sgACC to pACC, from striatum to pACC, from pACC to dlPFC, from striatum to sgACC, from sgACC to dlPFC, and from striatum to dlPFC), while the six panels in the bottom-left corner (red shaded area) show the top-down influences (GCIs from pACC to sgACC, from pACC to striatum, from sgACC to striatum, from dlPFC to pACC, from dlPFC to sgACC, and from dlPFC to striatum). **b,** The GCIs between pairs of different regions in the *Stim-on* block, with panels illustrated similarly to **a**. **c,** The GCIs in the *Follow-up* block after the *Stim-on* block, with panels illustrated similarly to **a**.

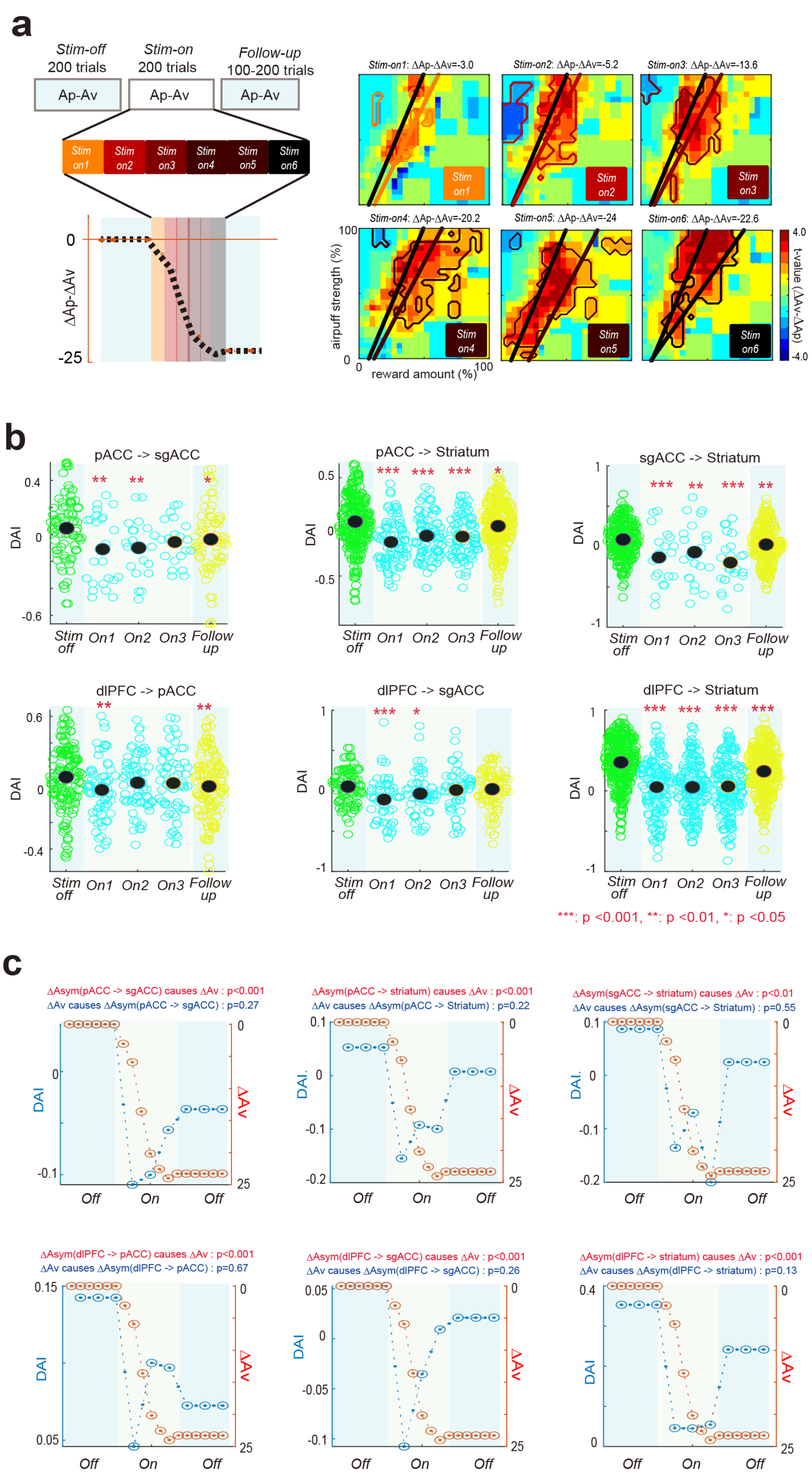
a
Stim-off
200 trials
Stim-on
200 trials
Follow-up
100-200 trials
Ap-Av
Ap-Av
Ap-Av
Stim on1
Stim on2
Stim on3
Stim on4
Stim on5
Stim on6
0
-25
ΔAp-ΔAv
Stim-on1: ΔAp-ΔAv=-3.0
Stim-on2: ΔAp-ΔAv=-5.2
Stim-on3: ΔAp-ΔAv=-13.6
Stim-on4: ΔAp-ΔAv=-20.2
Stim-on5: ΔAp-ΔAv=-24
Stim-on6: ΔAp-ΔAv=-22.6
airpuff strength (%)
reward amount (%)
t-value (ΔAv-ΔAp)
b
pACC -> sgACC
pACC -> Striatum
sgACC -> Striatum
dlPFC -> pACC
dlPFC -> sgACC
dlPFC -> Striatum
DAI
Stim off
On1
On2
On3
Follow up
***: p <0.001, **: p <0.01, *: p <0.05
c
ΔAsym(pACC -> sgACC) causes ΔAv : p<0.001
ΔAv causes ΔAsym(pACC -> sgACC) : p=0.27
ΔAsym(pACC -> striatum) causes ΔAv : p<0.001
ΔAv causes ΔAsym(pACC -> Striatum) : p=0.22
ΔAsym(sgACC -> striatum) causes ΔAv : p<0.01
ΔAv causes ΔAsym(sgACC -> Striatum) : p=0.55
ΔAsym(dlPFC -> pACC) causes ΔAv : p<0.001
ΔAv causes ΔAsym(dlPFC -> pACC) : p=0.67
ΔAsym(dlPFC -> sgACC) causes ΔAv : p<0.001
ΔAv causes ΔAsym(dlPFC -> sgACC) : p=0.26
ΔAsym(dlPFC -> striatum) causes ΔAv : p<0.001
ΔAv causes ΔAsym(dlPFC -> striatum) : p=0.13
DAI
ΔAv
Off
On
Off

Supplementary Fig. 13. Granger causality analyses between ∆DAI and ∆Av during the *Stim-on* block.

a, The sgACC microstimulation induced a cumulative increase in avoidance choices throughout the *Stim-on* block. We divided the *Stim-on* block into six periods (*Stim-on1*, ..., *Stim-on6*), and the behavioral choice patterns of the ten effective sessions were aggregated during each period (upper left panel) after the standardization procedure (see Methods). After applying spatial smoothing, the choice patterns during each period (*Stim-on1*, ..., *Stim-on6*) were compared with those during the *Stim-off* block (right panel) (see Methods). These analyses exhibited significant increases in Av and Ap for each combination of the reward and airpuff offers. The size of the increase in Ap (i.e., ∆Ap) and Av (i.e., ∆Av) were calculated for each period (i.e., *Stim-on1*, ..., *Stim-on6*). Finally, we calculated ∆Ap-∆Av to derive the temporal pattern of the behavioral changes (lower left panel). **b,** The changes in the Directional Asymmetry Index (DAI) during the *Stim-on* block. After removing artifacts (see Methods), the *Stim-on* block was divided into three periods (i.e., *On1*, *On2*, and *On3*). The DAI in the 5-30Hz range was calculated between two regions for each channel. The distributions of DAI for each period were compared with that in the *Stim-off* block. Further, the distribution of DAI during the Follow-up block was also calculated and compared with the DAI distribution during the *Stim-off* period (* $P < 0.05$, ** $P < 0.01$, *** $P < 0.001$, t-test). **c,** Granger causality analyses between ∆DAI and ∆Av during the *Stim-on* block. After performing interpolation for the time course of DAI, we compared the time courses of DAI and ∆Ap-∆Av for each combination of regions. In all combinations, the change in DAI temporarily led to ∆Ap-∆Av. Granger causality analyses estimated that ∆DAI Granger-caused ∆Ap-∆Av, while the reverse causality was not observed for all combinations of regions.

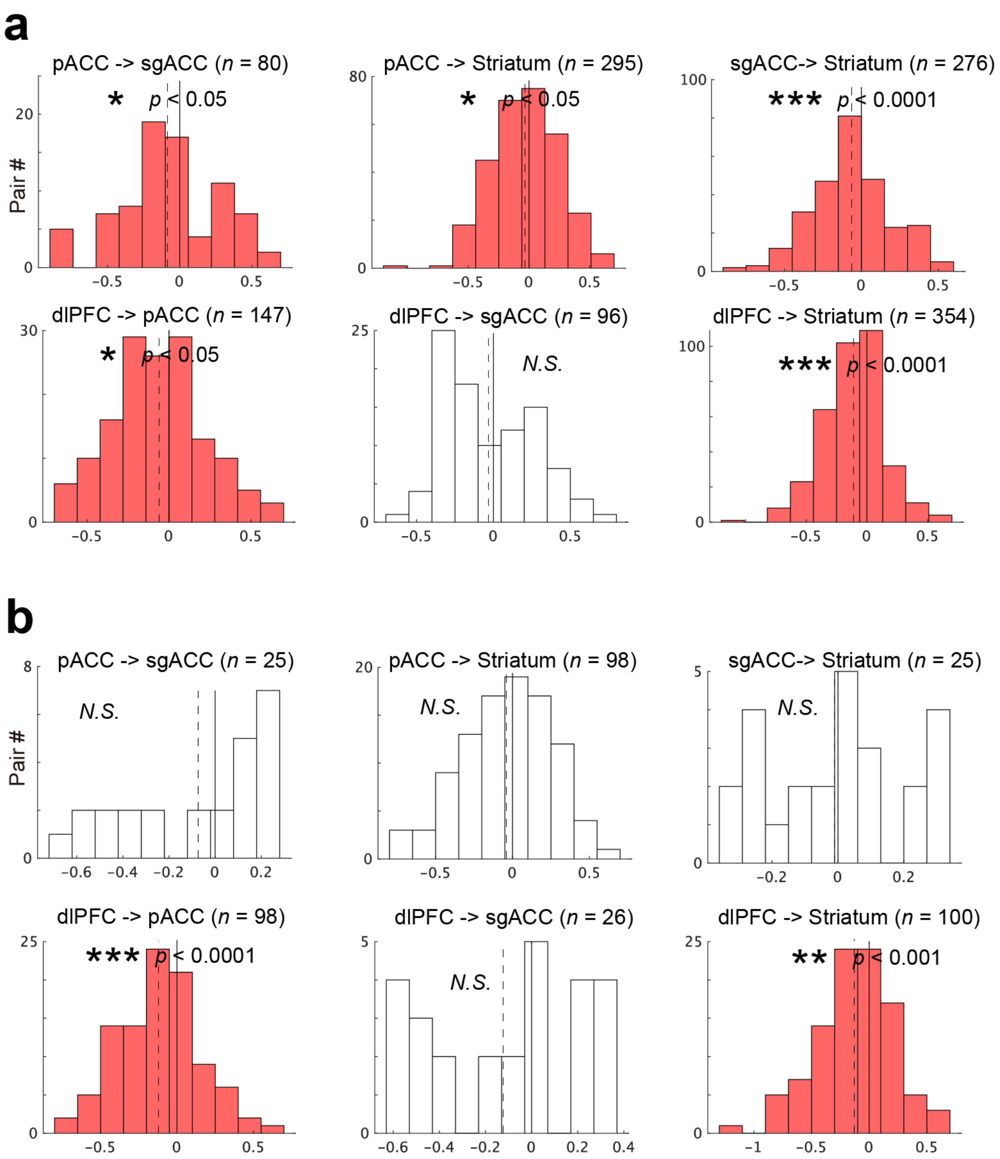

Supplementary Fig. 14. Distributions of the change in DAI for two monkeys.

a, Change in DAI (directional asymmetry index) for monkey P. DAI was calculated for each pair of brain regions and averaged over the alpha and beta frequency bands. The change in DAI was calculated as the difference between the mean DAI during the *Follow-up* block and the mean DAI during the *Stim-off* block. The black dotted line represents the mean of the distribution, and the red distribution indicates a significant deviation from zero (* $P < 0.05$, ** $P < 0.01$, *** $P < 0.001$, t-test). **b**, Change in DAI for monkey S. Each panel is illustrated as in **a**.

Supplementary Discussion

The accumulative effect of microstimulation.

The effect of microstimulation on the decision-making process is characterized by an accumulative change in value judgment. In analyzing ten effective sessions, we segmented the *Stim-on* block into six temporal periods (*Stim-on1*, ..., *Stim-on6*). Choice data for each period were aggregated across the ten effective sessions following the standardization procedure (see Methods). We assessed the difference in choice patterns between *Stim-off* and each of the six temporal periods. Notably, we observed a temporal accumulation of the stimulation effect on behavioral changes, reaching peak significance in the later stages of the stimulation block (Supplementary Fig. 13a). These characteristics align with findings from our prior studies on pACC[2] and striatal[1] microstimulation, indicating a robust pattern across various microstimulation experiments. The results imply a gradual modulation of reward-punishment weighting by sgACC microstimulation, with no direct influence on Ap-Av decisions. Examining the direct influence on the Ap-Av decision-making required a randomized order of stimulation on and off trials. Our previous work[2], involved such randomization, revealing no substantial changes in Ap-Av decisions. However, we did observe alterations in the slope of the decision boundary instead (Figure 7d of Amemori and Graibiel[2]). Based on these results, we concluded that sgACC microstimulation did not influence the decision-making process but might influence the neural plasticity of the value judgment.

Supplementary References

1. Amemori K, Amemori S, Gibson DJ, Graybiel AM. Striatal microstimulation induces persistent and repetitive negative decision-making predicted by striatal beta-band oscillation. *Neuron* **99**, 829-841 (2018).

2. Amemori K, Graybiel AM. Localized microstimulation of primate pregenual cingulate cortex induces negative decision-making. *Nature Neuroscience* **15**, 776-785 (2012).
